# Introduction of the Hepatitis B Vaccine—Birth Dose: Methods of Improving Rates in a Milieu of Vaccine Hesitancy

**DOI:** 10.3390/vaccines12010025

**Published:** 2023-12-25

**Authors:** Shivon Belle Jarvis, Tessy Fenton-Lee, Sinéad Small

**Affiliations:** Sir Lester Bird Medical Centre, Michael’s Mount, St. John’s, Antigua and Barbuda; tessy.fenton@msjmc.org (T.F.-L.); sinead.small@msjmc.org (S.S.)

**Keywords:** Antigua and Barbuda, hepatitis B, vaccination coverage, birth dose, elimination

## Abstract

The hepatitis B virus is a public health threat, chronically infecting over 240 million persons worldwide. The hepatitis B vaccine is 90% effective in preventing perinatal transmission if the first dose is given within the first 24 h of life, followed by a minimum of two subsequent doses. Antigua and Barbuda instituted a hospital-based birth dose vaccination policy in October 2021. Data were extracted from hospital logbooks from November 2021 to October 2022, and a database was created. Frequency distributions of the hepatitis B birth dose, barriers to administration, and maternal and healthcare system factors were analyzed. The positive maternal HBsAg prevalence rate was 0.6%. The timely and total birth dose coverage was 72% and 81%, respectively. In total, 10.5% of parents refused the vaccine, of which 76% either felt uncomfortable or preferred to wait. Moreover, 100% of hepatitis B-exposed babies were vaccinated, with 83% of them receiving the Hepatitis B Immunoglobulin. Barriers to vaccine administration included vaccination hesitancy, gaps in knowledge of medical staff, and the inconsistent vaccination supply. Instituting a quality improvement team, health information system, robust educational efforts, and addressing barriers will make achieving the WHO programmatic targets of eliminating mother-to-child transmission of hepatitis B by 2030 possible.

## 1. Introduction

Hepatitis B is a serious public health threat, with the hepatitis B virus (HBV) continually contributing to the global burden of disease [1,2]. This is responsible for most of the global hepatitis burden when compared to its counterparts: hepatitis A, C, D and E [3].

HBV, the second most important known human carcinogen, chronically infects over 240 million persons worldwide [4]. This can be transmitted vertically and horizontally and can ultimately result in premature death from cancer or liver cirrhosis. The World Health Organization (WHO) estimates that 4 to 5 million lives globally each year are saved because of vaccination, and the hepatitis B vaccine is no exception [5]. It not only protects against HBV infection in adults and children but is 90% effective in preventing perinatal transmission if the first dose is given within the first 24 h of life, followed by a minimum of 2 subsequent doses [4].

Development of chronic disease can be predicted based on age of acquisition of the HBV, with a risk of 90% if acquired in the perinatal period, 30–60% in early childhood, 5–10% in ages 5–20 years and 1–5% in adults over 20 years [6].

The elimination of mother-to-child transmission (EMTCT) plus initiative has, within its framework, steps to eliminate not only Human Immunodeficiency Virus (HIV) and Syphilis vertical transmission, but also HBV and Chagas infections. In December 2017, Antigua and Barbuda was validated by the World Health Organization Validation Advisory Committee (GVAC) as having eliminated mother-to-child transmission of HIV and Syphilis, with validation status being maintained in 2019 and 2020 and when last reviewed in 2022, as was communicated via email [7,8]. The EMTCT plus initiative is, therefore, the next step for the twin-island state with particular emphasis on triple elimination (HIV, Syphilis and HBV). At the Sir Lester Bird Medical Centre from 2020 to 2022, 26 mothers tested positive for hepatitis B surface antigen (HBsAg) of the 3160 mothers who had live births in the aforementioned years, which is equivalent to a prevalence rate of 0.8% [9].

Chronic hepatitis B infections have a prevalence rate of 0.33% (0.26–0.95%) in Latin America and the Caribbean, affecting 2.1 million persons, of which more than 13,000 deaths are estimated to occur annually from HBV and related diseases [10]. Perinatal transmission characterized 56% of the cases in 2016, therefore, important strides must be made not only in ensuring universal hepatitis B vaccination coverage but also in instituting a universal birth dose policy [10].

The World Health Assembly in 2016 endorsed the elimination of viral hepatitis as a public health threat to include elimination of mother-to-child transmission of hepatitis B by 2030 [11]. Validation of the latter through the WHO, for countries that provide universal hepatitis B vaccine at birth, requires ≥90% coverage with the birth dose and ≥90% coverage with three doses of the hepatitis B vaccine (HepB3) in infants, with maintenance of these targets for at least 2 years [12]. The impact of these targets is also monitored in order to achieve validation, which means that countries should achieve a ≤0.1% HBsAg prevalence among children ≤5 years [12]. The local framework for determining and tracking the HBsAg of children ≤5 years old remains in its embryonic stage and requires further discussion and planning.

Antigua and Barbuda, a twin-island state in the Caribbean, joined St. Kitts and Nevis, St. Vincent and the Grenadines, St. Lucia, Dominica and Grenada in instituting a universal hepatitis B vaccine birth dose (HepB-BD) program [13]. With a population of 97,928, Antigua and Barbuda has one primary public hospital, the Sir Lester Bird Medical Centre (SLBMC), with just over 1000 deliveries annually that offers both routine and critical newborn care, managing neonates of gestational ages of 26 weeks and above [14]. As the country’s primary facility, it accounts for over 97% of births, with only one private facility at the time of publication offering delivery services [15]. In 2021, the SLBMC accounted for 99.5% of the nation’s live births. The HepB-BD, was introduced on 11 October 2021 at the Sir Lester Bird Medical Centre. The vaccine is procured by the government of Antigua and Barbuda through the Pan American Health Organization’s Revolving Fund and through the Global Alliance for Vaccines and Immunizations. Vaccines are offered to the public free of cost. Prior to the program’s formal introduction, it was ensured that an institutional policy was prepared and implemented with the necessary logistics put in place regarding vaccination storage and logging post administration. Staff were then oriented to the policy and trained on key topics. These included the rationale for the HepB-BD, the crucial importance of timing administration and documentation, the revised immunization schedule, the importance of maintaining the cold chain and the quality assurance steps that would be implemented. Staff training took place within the hospital and primary healthcare facilities and included public and private healthcare providers. This facilitated the distribution of healthcare worker educational guides. Client education was primarily facilitated on an individualized basis during antenatal visits, given the marked misinformation that already existed surrounding the COVID-19 vaccine, leading to a general milieu of vaccine hesitancy. Mass media educational drives were avoided. A client brochure was also prepared to better enable the educational process. This was given to the client either during their antenatal visit or while at the birthing facility.

The national Maternal Child and Adolescent policy manual offers guidance that all pregnant women should have their HBsAg status assessed and documented in the first and third trimester, which enables the appropriate management to be instituted antenatally and postnatally [16].

### 1.1. Definitions [4]

1. Timely birth dose coverage: the proportion of live births who receive a HepB-BD within 24 h after birth. All doses given on day 0 or 1 of life meet this definition, i.e., the date of delivery and the day following delivery. This is the global standard for monitoring HepB-BD coverage.
2. Total birth dose coverage: the proportion of live births who receive any HepB-BD, defined as those vaccinated any time up until the first dose is due, or as per country guidance on upper age range, i.e., 14 days.
3. Health facility birth dose coverage: the proportion of live births in a health facility who receive HepB-BD. This may be tracked separately if there are different coverage targets for facility births, especially regarding timely birth dose administration.
4. Home birth dose coverage: the proportion of live home births who receive HepB-BD. This section outlines the methods for monitoring and evaluating a HepB-BD vaccination.

The denominator used for calculating coverage was the number of live births.

### 1.2. Rationale

Given the rapid startup of the HepB-BD program in a vaccine-hesitant milieu and the absence of a mass media educational drive, there were likely quality gaps that existed, which would negatively impact vaccine uptake and timely administration. A review would, therefore, seek to establish vaccination rates and outline quality improvement steps that can be taken in order to improve indicators of interest.

### 1.3. Objectives

1. Review the timeliness of HepB-BD administration at the SLBMC;
2. Review the uptake of the HepB-BD at the SLBMC, capturing the reasons for refusal;
3. Outline recommendations for quality improvement for the HepB-BD program.

### 1.4. Research in Context

Medline, PubMed, Semantic scholar and Cochrane databases were explored using the following key words: hepatitis b vaccine, birth dose, elimination of mother to child transmission, universal. MeSH terms Hepatitis B vaccine and Birth dose were utilized. The search was not limited by article type, but articles within a customized range of 15 years were used. Only Articles written in English were utilized.

### 1.5. Added Value

This manuscript highlights an added country on the path to triple elimination. By highlighting barriers faced and successes in a Caribbean context and clearly stating useful recommendations, this may aid other jurisdictions as they implement or strive to improve the universal newborn hepatitis B vaccine program. This will also help to improve the quality of current programs as well, and inform policy.

## 2. Materials and Methods

### 2.1. Administration of the HepB-BD by the Healthcare Worker

Prior to the administration of the HepB-BD, verbal consent was attained from either mother or father by the healthcare worker, most commonly, the nurse midwife. This was usually attained postdelivery. The consent from mothers or fathers who were minors, i.e., anyone under the age of 16 years, for the administration of the HepB-BD to their baby was primarily done in the presence of their guardians. However, this was not formally addressed in the hospital’s vaccination policy [17]. Once the vaccine was administered, data including maternal HBsAg status, date and time of birth, date and time of administration of the vaccine, batch number and expiration date were captured in a log book. This was kept in a locked cupboard on the maternity ward.

If the vaccine was refused, the reason for refusal was documented in the newborn’s chart. This would then be noted when the newborn was subsequently seen by the clinician. If the decision of refusal remained unchanged post educational efforts by the physician, then data void of patient identifying details were added to a refusal list on the maternity ward, within the clinician’s purview to include the reason for refusal of the vaccine.

The aforementioned processes were as outlined within the Hospital HepB-BD vaccination policy. Notably, documentation of the maternal HBsAg status within the log book formed a part of the disease control measures implemented at the SLBMC. This book enabled a clinician to quickly capture women who were positive and allowed tracking of the timing and administration of the Hepatitis B Immunoglobulin (HBIG) to exposed neonates. This recording mechanism was, therefore, formally a part of the infection control monitoring mechanism. Maternal consent was, therefore, not required for documentation of this status within an established hospital recording system.

### 2.2. Data Collection

As this was an audit of an implemented hospital program, a database was created through the use of Excel. A data extraction sheet void of patient-identifying information was created to include the following fields: maternal age, maternal HBsAg status, route of delivery, birthweight, neonate’s date and time of birth, day, date and time of administration of the HepB-BD, facility/location of birth whether SLBMC or BBA, if the birth dose was refused, reason for refusal, and if indicated whether the HBIG was administered to include date and time of administration. A data field was also included labeled ‘Criteria not met’. Data were entered into this field if a baby was identified in the log book with a birth weight of <2000 g and a maternal HBsAg result that was negative.

Data were retrospectively extracted at the end of each month from the HepB-BD log book and the maternity log book. The total number of vaccines administered and refused was reviewed and then compared to the number of live births from the maternity log book of births and deaths. This enabled capturing any missed doses. It is important to note that maternal age and route of delivery were specifically extracted from the maternity ward’s log book. Babies born at home or before arrival to the hospital were also included in the database once they presented to SLBMC for care within 24 h of delivery and were documented within the aforementioned log books. The reason for refusal was extracted at the end of each month from the refusal list stored on the maternity ward. Data were collected from November 2021 to October 2022.

Inclusion Criteria: Newborns delivered at SLBMC

- Born before arrival to SLBMC presenting to this institution within 24 h of delivery.

Exclusion criteria: Babies born at another birthing facility (i.e., outside of SLBMC)

- Babies born at home not presenting to SLBMC.

### 2.3. Data Analysis

Data are presented as frequencies and percentages, with the primary measure being captured as the proportion of live births who received the HepB-BD within 24 h of birth, and the secondary measure captured as the proportion of livebirths who received the HepB-BD within 14 days of life as per country protocol. The odds ratios and their confidence intervals were also calculated and used to estimate the possible association of factors of interest with outcome. Data analysis was done using Excel 2019 and Stata v.18.

### 2.4. Ethics

As this was an audit of existing hospital records, the current study was considered exempt from the need for approval by SLBMC’s IRB. Ethical Approval was attained from the MOHWE IRB.

Patient interviews were not conducted, therefore, there was no direct contact with the clients. The audited materials were the HepB-BD and maternity log books, and the refusal list. Since the HepB-BD log book had patient-identifiable information, it was stored in the unit manager’s office in a locked cupboard. The data collected and processed, as outlined in the indicators above, had no patient-identifying information. The data extraction Excel sheet and Stata v. 18 file were stored in a password-protected computer on the maternity ward and will be kept for 2 years.

## 3. Results

**Figure 1:**
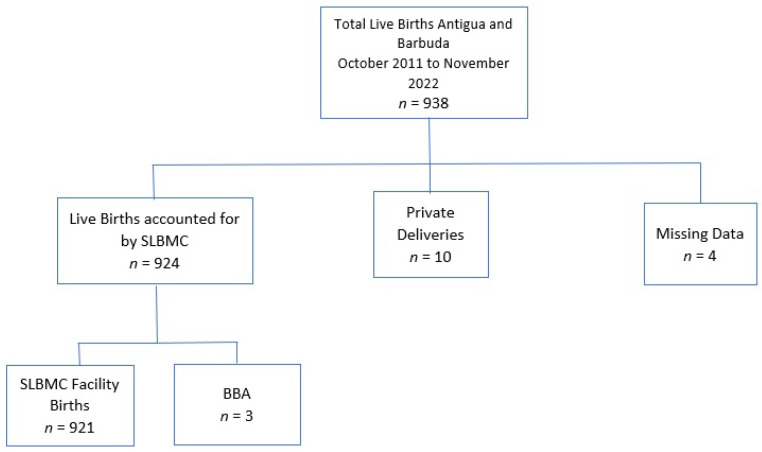
Flow chart demonstrating distribution of live Births in Antigua and Barbuda October 2011–November 2022.

The study period of November 2021 to October 2022 was characterized by 924 live births being accounted for by the SLBMC, with an average of 77 deliveries per month (95% CI 67.6, 86.4). This represented 98.5% of national live births (*n* = 938) over the study period (see flow chart in Figure 1) [18]. Further analysis revealed that 99.7% of babies at SLBMC were facility-born, and 0.3% were born before arrival (BBA) to hospital but subsequently presented for care at the SLBMC within 24 h of life. Of the live births at SLBMC, 4.3% (*n* = 40) weighed less than 2000 g. The mean maternal age was 28 years (95% CI 27.82, 28.67), the youngest being 11 years, the eldest being 45 years.

**Figure 2:**
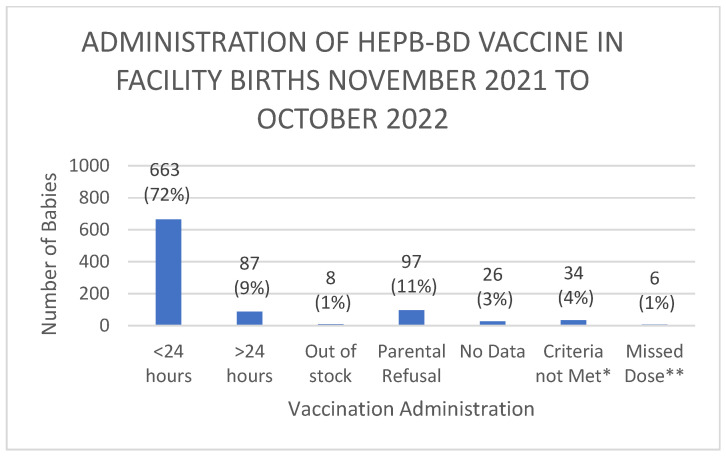
Breakdown of HepB-BD administration in babies born at SLBMC. * Criteria not met—weight < 2000 g, maternal hepatitis B surface antigen (HBsAg) negative, therefore, vaccine not administered. ** Missed Dose—includes babies <2000 g whose maternal hepatitis B status is unknown (*n* = 6).

Of the babies born at the SLBMC, i.e., facility births, further details regarding vaccine availability and administration are demonstrated in Figure 2 below.

Notably, 100% of babies BBA received the vaccine within <24 h of birth. Overall, 72% of live births received timely administration of the HepB-BD, whilst total birth dose coverage was 81% of live births. The maximum duration of time from birth to vaccination administration was 11 days 4 h and 55 min.

### 3.1. Parental Refusal

**Figure 3:**
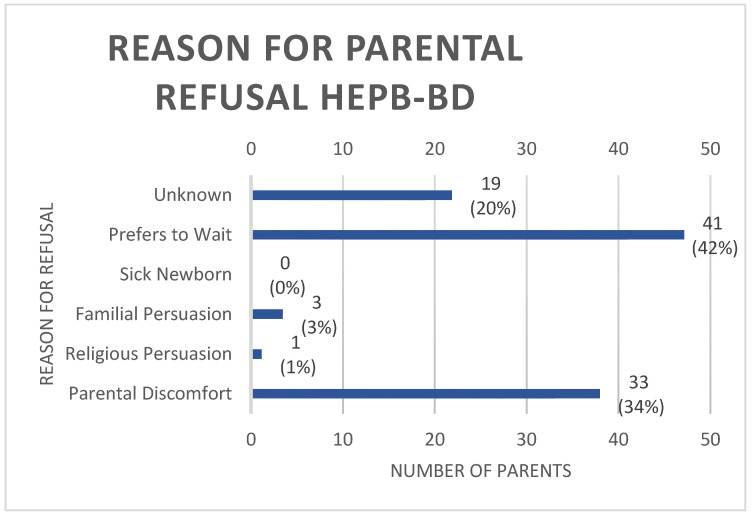
Rationale for parental refusal of HepB-BD.

Parental consent was routinely attained by the physician or nursing staff, as per policy, prior to the administration of the HepB-BD. In total, 10.5% of parents refused the HepB-BD. Issues raised at the time of consent that impacted refusal were classified and documented as follows: parental discomfort with the vaccine, including lack of sufficient knowledge, familial persuasion against vaccine administration, having a sick newborn, religious persuasion, unknown reason and the desire to wait until 2 months of age. Figure 3 demonstrates the aforementioned reasons for refusal.

### 3.2. Maternal Hepatitis B Status

The maternal HBsAg status was reviewed prior to the vaccination of the newborn. It is important to note that the maternal status captured was at the time of vaccination administration and does not reflect maternal results that were subsequently followed. A documented maternal HBsAg result was available for 90.4% of mothers of this cohort. The prevalence rate for a positive maternal HBsAg was 0.6%. Table 1 demonstrates the HepB-BD receipt frequency by maternal HBsAg status.

A positive maternal HBsAg result triggered the administration of both the HepB-BD and the HBIG. Of the six babies born to HBsAg-positive mothers, all received the HepB-BD—one baby in just over 24 h and all others in <12 h. In 83% (5/6) of the exposed newborns, the HBIG was administered in <12 h, whilst one exposed baby was unable to receive the Immunoglobulin as it was unavailable.

### 3.3. Factors Influencing Administration

**Figure 4:**
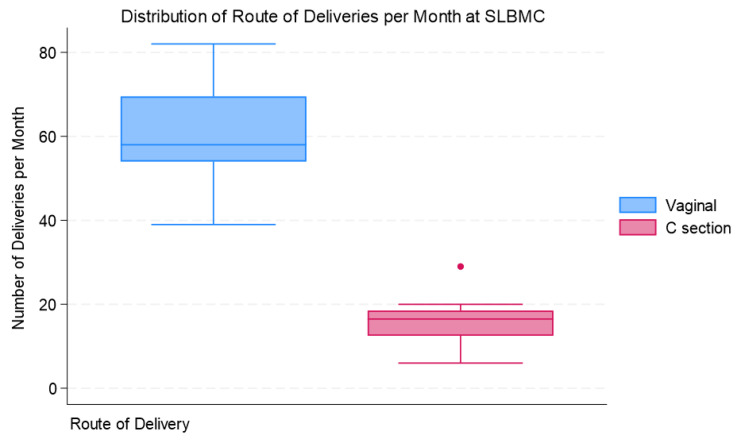
Distribution of route of deliveries per month at SLBMC.

Factors associated with the administration of the vaccine are demonstrated in Table 2 below. Babies born to mothers <35 years of age who received timely administration of the vaccine had an odds ratio of 1.14 times that of babies born to mothers ≥35 years-old who received the vaccine in a timely manner. Note should be made, however, that other confounders were not controlled for to include maternal highest educational level, parity, and socio-economic status. This could be addressed through design adjustments to include randomization, restriction or the establishment a selection criterion. There was no statistically significant difference in the odds of receiving the vaccine in a timely manner on the weekend or in the week. Babies born via vaginal delivery who received the vaccine in a timely manner had an odds ratio that was 2.508 times that of babies born via C-section who received a timely dose; this was statistically significant. It should be noted, however, that vaginal delivery is the primary route of delivery (79% of deliveries) and, therefore, could contribute to confounding error. The box plot in Figure 4 demonstrates the distribution of the monthly delivery routes. The median number of vaginal deliveries per month was 58 with an interquartile range of 15.5 deliveries. The median number of Caesarean sections was 16.5 deliveries per month, with an interquartile range of six deliveries per month.

In a review ”Factors associated with receipt of a timely infant birth dose of hepatitis B vaccine at a tertiary hospital in North-Central Nigeria” by Bada et al. (2022), maternal factors, such as parity, marital status or educational status, had no significant impact on the timely administration of the HepB-BD [19].

A review of the processes that govern the administration of the HepB-BD post a C-section delivery, however, is warranted, as the timing of consent can potentially be delayed due to recovery time and other competing priorities that may occur immediately post C-section.

## 4. Discussion

### 4.1. Coverage and Timeliness

There has been progress globally in the fight of EMTCT of hepatitis B as >98% of World Health Assembly member states have introduced a universal infant hepatitis B vaccination program [11]. Despite this, the introduction of the HepB-BD into routine immunization programs has much to be desired. In 2020, only 57% of these countries provided the HepB-BD routinely to all newborns, with its timely administration ranging from 37 to 43% from 2016 to 2020 [13]. The Sir Lester Bird Medical Centre, having achieved a timely birth dose of 72% during the time period of this audit, is commendable. However, more work is required to achieve the 2021 programmatic target of WHO of having ≥90% timely HepB-BD national immunization coverage. The total birth dose coverage at the time of this audit was 81%. However, from 2016 to 2020, of the global countries that reported, 51–58% of them confirmed a HepB-BD coverage rate of >90% [11]. Canada has not completely embraced the recommendation of WHO for universal administration of HepB-BD, as only three provinces and territories observe this public health practice [20]. The catalyst for this decision was based on the assumption that there is universal prenatal screening for HBV, although this was found to be imperfect, that postexposure prophylaxis was administered to all babies born to HBsAg-positive mothers, and that waning protection after infant vaccination could leave the adult population predisposed [20]. The program in the UK also remains a selective program with the at risk/exposed newborns being targeted. The Joint Committee on Vaccination and Immunization (JCVI) did not find it cost-effective as most chronic hepatitis in adults is imported, thus making a universal newborn vaccination program an academic debate [21]. They did, however, establish >90% coverage of the HepB-BD in the exposed newborns [21]. With the highest prevalence of chronic hepatitis B in the world, Africa represents two of every three children under the age of 5 years globally with chronic HBV. Birth dose coverage, however, remains inconsistent and was 17% in 2021 for the WHO African region [22]. Nigeria had a coverage rate of 53% [23]. Pelts and Lemma (2020), in an AAP national conference and exhibition meeting where abstracts were reviewed, reported that over a 2-year quality improvement program, HepB-BD coverage rate improved from 65% to 69% and that timely administration improved to 43% [24]. In a national survey, Myanmar, from 2011 to 2016, had a HepB-BD coverage rate of 26% [25]. The birth coverage rates, as well as the timely administration of the HepB-BD in Antigua and Barbuda at the SLBMC are, therefore, exemplary when compared to other territories, as discussed and demonstrated in Table 3. It is understood, however, that each territory actively faces different barriers and that working with one facility with a smaller sample size may better enable coverage and timely administration of the vaccine at birth.

The HepB-B3 coverage of >90% is also a crucial target. Although it is not formally reviewed in this audit, it is necessary to mention. In 2021, in the Region of the Americas, the HepB3 coverage in children under the age of 1 was 81%, with Antigua and Barbuda reporting 92% coverage [26,27]. Table 3 below aptly reflects timely birth coverage and HepB3 coverage for various regions. Comparing these coverage rates to immunizations with similar schedules can help to put things into context. As of 2022, the coverage rate of the third dose of Hemophilus Influenza type B in Antigua and Barbuda was 99% [28].

### 4.2. Maternal HBsAg Prevalence Rates

The local maternal HBsAg prevalence was 0.6% during this study period, compared to a rate of 0.8% from 2020 to 2022. This was similar to the HBsAg prevalence of 0.6% in pregnant women in Ontario, Canada, from 2012 to 2016, and the UK with a 0.4% prevalence rate of chronic HBV in pregnancy [20,21]. On the contrary, Myanmar has an HBV seroprevalence of 8.5%, with prevalence rates in Central and East African countries varying to include “3.2% in Eritrea, 9.3% in Kenya, 3.1% in Kigali, and 7.5% in Sudan” [25,29]. Higher rates impact the burden of disease and continue to strengthen the appeal for EMTCT through various mechanisms to include universal HepB-BD administration, which has the potential of reducing seroprevalence rates in children. This, therefore, indicates the importance of reviewing the HBV seroprevalence of children under the age of 5 years as a validation measure of coverage and reliability of the cold chain. Table 4 demonstrates the prevalence of HBV in pediatric cohorts before and after the implementation of HepB-BD, thus emphasizing the apparent impact of the hepatitis B universal birth dose implementation.

### 4.3. Barriers/Education

#### 4.3.1. Client Level

Parental hesitancy remains a crucial factor that needs to be addressed. Of the parents who refused the vaccine, 76% either felt uncomfortable or preferred to wait. The legal right of parents to refuse vaccines must always be considered. However, further exploration of these concerns is vital in informing future educational efforts. These programs need to be more robust, capturing not only expectant families during the antenatal period but also their primary support system. They may include public service announcements, WhatsApp blasts, social media campaigns, town halls, a hotline, discussions in antenatal classes, radio and TV programs and newspaper articles that can clearly state facts and address common misconceptions. The power of community-based educational initiatives should not be overlooked [30]. The strengths of this program include the availability of pamphlets for families as well as the availability of a staff handouts that serve as a standard guide to answering frequently asked questions. It is recognized that local experience can be used to guide strategy, as that which is given by WHO serves as a starting point [30]. The information that transpired in neighboring and comparable settings should also be reviewed and used as a guide [30].

Three factors are included in the WHO vaccine hesitancy model: confidence (in vaccine or provider), complacency (need for and value of vaccine not appreciated), and convenience (barriers to access to vaccines) [31]. Lack of confidence and complacency are likely two factors that impacted our program.

The milieu at the time of the roll-out of this program in 2021 should be considered. The COVID-19 pandemic has generally changed parents’ intention to vaccinate their children [30]. COVID-19 was thought to have a heterogenous impact on vaccination in countries across the region, with 15 countries in 2020 and 2021 reporting their lowest HepB-B3 coverage in the previous 10 years [13,26]. That compounded with the historical fact that in Antigua and Barbuda, the HepB-Bd was only selectively given to the hepatitis B-exposed infants with first vaccines, otherwise being administered at 2 months of age, could have influenced one’s desire to wait.

Parental refusal is a barrier experienced in other jurisdictions. Alternate vaccination schedules readily available online and emphasis on legal rights continue to strengthen their persuasion [24]. In Myanmar, women who had weekly media exposure and lived in the wealthiest households in urban areas, with the receipt of early and adequate antenatal care (≥8 visits) were more likely to have babies who received the HepB-BD [24]. These are factors that can be reviewed locally in the future. Lack of parental health literacy regarding the impact of hepatitis B on the newborn, maternal denial of her positive hepatitis B status, cost of the birth dose and the HBIG, and the partners’ noninvolvement post-testing were additional factors identified in Ghana [32]. Although the HepB-BD is free of cost in Antigua and Barbuda, there is a cost associated with receiving the HBIG, which is an out-of-pocket cost covered by the client. This was not a limiting factor locally, as once the HBIG was available, it was received.

Transitioning to having a standing order instituted where the vaccine is not given if there is a medical rationale or where the parent ‘opts out’ may be a step that needs to be considered. In the Philippines, hospitals with a standing order were 4.8 (CI 1.2–18) times more likely to have more than 50% coverage. The findings, however, were limited as no control for confounders was made [33].

#### 4.3.2. Facility Level

##### Knowledge of Healthcare Workers

Staff training and having a written policy were assets to this program. However, there were missed opportunities for vaccine administration. A gap identified is the vaccination of babies <2000 g whose maternal HBsAg status was unknown. Moturi et al. (2018), in reviewing the knowledge, attitudes and practices regarding the implementation of the birth dose of the hepatitis B vaccine in five African countries, found that most staff had suboptimal knowledge regarding the age limits and contraindications of the HepB-BD [34]. Staff training was only conducted prior to the initiation of the HepB-BD vaccination program. This was a limitation of the program at SLBMC. Conducting refresher training courses that incorporate the gaps identified is useful and should include healthcare professionals in public and private practice [29]. Continuous data sharing regarding the new findings through monitoring and evaluation of the program is crucial. Training and orientating new staff are also an important step in maintaining the quality of the HepB-BD program.

##### Supply Chain

Antigua and Barbuda subscribes to GAVI and the PAHO revolving fund, which is a crucial step in ensuring a reduced likelihood of the interruption of the supply chain of vaccines. During the timeframe of this audit, 0.9% of babies were unable to receive the HepB-BD due to the vaccine being out of stock. Disruptions in the supply chain for vaccines of the WHO Expanded Program on Immunization due to the COVID-19 pandemic are concerning, even as they impact developing countries [35]. This concern continues in the postpandemic era.

##### Regulatory Considerations

Adverse health outcomes potentially related to HepB-BD administration should be continually monitored by the hospital’s surveillance unit with subsequent reporting to the surveillance unit for the MOHWE. This should be clearly outlined in the hospital and national policy so that the mechanism of reporting is clear [36].

## 5. Limitations

Although mention was made of the importance of staff training in optimizing timeliness and coverage of the HepB-BD, the number and categories of staff trained were not captured. Their knowledge prior to and post training, concerns or personal biases were not explored. This study was also limited by not exploring and adjusting for potential covariates that could impact the timeliness of vaccine administration. It would have been beneficial to explore the timeliness and coverage of the HepB-BD administration at a private birthing facility, though having a smaller number of deliveries so that the program of all birthing facilities within our twin-island state could have been explored.

## 6. Recommendations

Recommendations, therefore, include instituting innovative educational sessions that capture parental concerns in the local context, implementing recertification programs for staff, and a quality improvement team that reviews birth dose timeliness and coverage with monthly or quarterly targets in place. Utilizing a database/health information system will strengthen the monitoring and evaluation of the program. Formalizing a national policy that governs public hospitals and private institutions is crucial. This will help inform/regulate the process of reporting from both facilities to the MOHWE. A review of the hepatitis B seroprevalence in children under the age of 5 years will help to validate birth dose coverage and the cold chain reliability and should, therefore, be considered. The sustainability of this program is heavily dependent on leadership and governance; therefore, ensuring a consistent vaccine supply chain through regional and international partnerships and implementing the necessary framework that supports the monitoring of the established targets is fundamental. This should fall under the purview of the Maternal, Child and Adolescent Health Committee of the Ministry of Health, Wellness, the Environment and Social Transformation.

## 7. Conclusions

The Sir Lester Bird Medical Center has achieved a timely HepB-BD coverage of 72% and total birth dose coverage of 81% from November 2021 to October 2022. This is a commendable start within the first year of this vaccine’s roll-out despite the milieu of vaccine hesitancy. With continued improvement in client educational campaigns, staff refresher courses with emphasis on gaps identified, processes that govern administration post-Cesarean section and possible implementation of a standing order approach governing vaccination administration within 24 h post-delivery; then timely birth dose coverage and total birth dose coverage will likely improve. This will put the twin-island state in good stead as we journey toward EMTCT of hepatitis B.

## Figures and Tables

**Table 1:** HepB-BD frequency by maternal HBsAg status.

Maternal HBsAg Status	HepB-BD Received *n* (%)	HepB-BD Not Received *n* (%)	No Data *n* (%)	Total Live Births *n* (%)
Negative	677 (73.3)	129 (14.0)	23 (2.5)	829 (89.7)
Unknown	69 (7.5)	15 (1.6)	2 (0.2)	86 (9.3)
Positive	6 (0.6)	0	0	6 (0.6)
No Data	1 (0.1)	1 (0.1)	1(0.1)	3 (0.4)
Total	753 (81.5)	145 (15.7)	26 (3.0)	924

**Table 2:** Factors associated with timely versus untimely administration of the HepB-BD.

Characteristic	All	Timely Administration	Untimely Administration	OR (95%CI)	*p* Value
Maternal age < 35	742	545	62	1.140 (0.995–1.307)	<0.05
Maternal age ≥ 35	182	118	24		
Weekend	212	193	21	1.192 (0.807–1.761)	>0.05
Weekday	538	470	65		
Vaginal	730	574	29	2.508 (1.865–3.372)	<0.05
C-section	194	89	55		

**Table 3:** Coverage for timely hepatitis B birth dose and HepB3 infant vaccine by WHO Region [22].

WHO Region	HepB-BD Coverage (%)	HepB3 Coverage (%)
African Region	17	71
Region of the Americas	59	80
Eastern Mediterranean Region	33	82
European Region	43	91
Southeast Asian Region	51	82
Western Pacific Region	78	90
Global Average	42	80

**Table 4:** Prevalence of HBV in pediatric cohorts pre- and postimplementation of HepB-BD [22].

Country	Hepatitis B Surface Antigen Positive (%)		Percentage Decrease in HBsAg Prevalence after HepB-BD Introduction (%)
	Before HepB-BD Introduction	After HepB-BD Introduction	
American Samoa	11.0	0.2	98
China	9.9	0.3	97
Malaysia	2.7	0.4	85
French Polynesia	3.0	0.0	99
Saudi Arabia	6.7	0.2	96
Singapore	4.2	0.4	93
Republic of Korea	8.0	0.2	98
Taiwan (Taipei)	9.8	0.7	93
Marshall Islands	12.0	0.6	95
Ornan	2.3	0.5	78
Thailand	4.5	0.6	87
United States (Alaska)	8.0	0.0	100

## Data Availability

The data presented in this study are available on request from the corresponding author. The data are not publicly available due to privacy restrictions.
